# Association of Plasminogen Activator Inhibitor-Type 1 (-675 4G/5G) Polymorphism with Pre-Eclampsia: Systematic Review

**DOI:** 10.1371/journal.pone.0056907

**Published:** 2013-02-15

**Authors:** Jessie A. Morgan, Sarah Bombell, William McGuire

**Affiliations:** 1 Hull York Medical School & NIHR Centre for Reviews and Dissemination,University of York, York, United Kingdom; 2 Department of Obstetrics, Goulburn Base Hospital, New South Wales, Australia; Queen's University, Canada

## Abstract

**Background and Aims:**

Excessive generation of plasminogen activator inhibitor-type 1 (PAI-1) is implicated in the pathogenesis of pre-eclampsia and related conditions. The PAI-1 (−675 4G/5G) promoter polymorphism (rs1799889) affects transcriptional activity and is a putative genetic risk factor for pre-eclampsia. The aim of this study was identify, appraise and synthesise the available evidence for the association of the PAI-1 (−675 4G/5G) polymorphism with pre-eclampsia.

**Methods:**

Systematic review and random effects meta-analysis of genetic association studies.

**Results:**

We found 12 eligible genetic association studies in which a total of 1511 women with pre-eclampsia, eclampsia or HELLP syndrome and 3492 controls participated. The studies were generally small (median number of cases 102, range 24 to 403) and underpowered to detect plausible association sizes. Meta-analysis of all of the studies detected statistically significant gene-disease associations in the recessive [pooled odds ratio 1.28 (95% confidence interval 1.09, 1.50); population attributable risk 7.7%] and dominant [pooled odds ratio 1.21 (95% confidence interval 1.01, 1.44); population attributable risk 13.7%] models. We did not find evidence of statistical heterogeneity, funnel plot asymmetry or small study bias.

**Conclusions:**

These data suggest that the fibrinolytic pathway regulated by the PAI-1 gene may contribute to the pathogenesis of pre-eclampsia and related conditions. This association, if confirmed in larger genetic association studies, may inform research efforts to develop novel interventions or help to prioritise therapeutic targets that merit evaluation in randomised clinical trials.

## Introduction

Coagulation and fibrinolytic cascades may be important components of the pathogenic process leading to pre-eclampsia, eclampsia or HELLP syndrome (microangiopathic haemolysis, thrombocytopaenia, and elevated liver enzymes) [1], [2]. One proposed mechanism is that excessive release of plasminogen activator inhibitor type 1 (PAI-1), a key down-regulator of endogenous fibrinolytic activity, from activated endothelium promotes spiral arterial or intervillous thrombosis that reduces placental perfusion [3], [4]. The reduction in blood flow to the placenta triggers the release of factors that further activate maternal vascular endothelium and culminates in the clinical entity of pre-eclampsia. This theory is supported by studies that have demonstrated higher plasma levels of PAI-1 in women with pre-eclampsia compared with gestation-matched pregnant women who are not hypertensive [5]–[8]. However, in defining the contribution of complex biochemical cascades to the pathogenesis of pre-eclampsia, it is difficult to distinguish molecular mechanisms that are causal from those that are epiphenomena of the disease process. It is possible that the finding of elevated plasma levels of PAI-1 in women with pre-eclampsia is secondary to endothelial damage (“reverse causation”) and does not indicate a causal role in the pathogenic process.

An approach that obviates this problem is to determine whether genetic polymorphisms that increase PAI-1 production are associated with the risk of developing pre-eclampsia. The most commonly studied functional variant in the PAI-1 gene is the guanine deletion polymorphism at position -675 nucleotides relative to the transcription start site (rs1799889). The PAI-1 (−675 4G) allele has higher transcriptional activity than the PAI-1 (−675 5G) allele and homozygous possession of −675 4G is associated with higher plasma PAI-1 levels [9], [10]. However, genetic epidemiology studies that have examined the association between the PAI-1 (−675 4G/5G) polymorphism and pre-eclampsia have reported conflicting findings but have been generally been too small to exclude plausible genotypic risks [11], [12]. Meta-analysis of data from several studies may provide more precise estimates of effect sizes. This approach has been used to define the size of the association of other putative genetic risk factors with pre-eclampsia [13], [14].

## Methods

We performed a systematic review and meta-analysis of genetic epidemiology studies of maternal carriage of the PAI-1 (−675 4G) polymorphism and pre-eclampsia and related conditions. We used methods recommended by the Human Genome Epidemiology Network [15]. We registered the study on PROSPERO, the international prospective register of systematic reviews (registration number CRD42012001904).

### Search strategy

We searched MEDLINE and EMBASE (1996–October 2012) via OVID using these terms:

1. [pregnan* AND (blood press* OR hypertens*)] OR PIH OR pre-eclampsia OR eclampsia OR pregnancy induced hypertension OR HELLP syndrome

AND

2. [Plasminogen Activator Inhibitor 1 OR PAI OR SERPINE] AND polymorphism.

We did not limit the search by language.

We cross-checked the MEDLINE “related articles” link in the PubMed interface for any potentially relevant studies. We also searched the US National Institutes of Health-sponsored Genetic Associations Database (http://geneticassociationdb.nih.gov) and the reference lists of all potentially eligible articles.

### Inclusion criteria

Case-control and cohort studies that assessed the association of the PAI-1 (−675 4G/5G) polymorphism with pre-eclampsia were eligible for inclusion provided that:

(1) Pre-eclampsia was defined as per international consensus criteria as systolic blood pressure ≥140 mm Hg or diastolic blood pressure ≥90 mm Hg occurring after 20 weeks' gestation in a woman whose blood pressure has previously been normal accompanied by proteinuria of ≥300 mg per 24 h or ≥”2+” by semi-quantitative near-patient testing (“dipstick”) [16]. Studies that included women with pre-existing or gestational hypertension without proteinuria were excluded. Studies that included women with eclampsia or HELLP syndrome were included.

(2) The control group consisted of women without a history of pre-eclampsia in pregnancy. Studies that used controls recruited from a general population (for example, blood donors) were excluded.

(3) Cases and controls were matched for ethnic group or the study reported the ethnic ancestry of participants to allow for stratified analysis.

(4) Genotype distribution within the controls was consistent with Hardy-Weinberg equilibrium assessed using Pearson's χ^2^ analysis [17].

Two authors independently used a standardised reporting form to abstract information on setting, design, and inclusion criteria from each potentially eligible study. We checked any studies by the same author(s) for possible overlapping participant groups. We contacted study investigators to obtain additional information if necessary.

### Data abstraction and quantitative synthesis

Two authors independently abstracted genotype data from each included study. We compared these results and resolved disagreements by consensus of three authors. We analysed genotype data by combining individuals homozygous and heterozygous for each of the two alleles into a single exposure class for cases and controls:

- Recessive model: 4G/4G versus [4G/5G or 5G/5G]
- Dominant model: [4G/5G or 4G/4G] versus 5G/5G

We used RevMan (version 5.1) to calculate an odds ratio (OR) with 95% confidence interval (CI) for each comparison in each study. We synthesised the data using the inverse variance-weighted method in a random effects model to calculate the summary OR and 95% CI. We determined the proportion of total variation contributed by between-study variation using the I^2^ statistic [18]. We assessed asymmetry in a funnel plot of effect size estimate versus precision to evaluate possible publication bias and used a linear-regression model to explore small study bias [19]. We undertook a post hoc subgroup analysis of the studies based on sample size of cases comparing the pooled OR of studies with < median number of cases versus those with ≥ median number. We used the method described by Altman and Bland to assess statistical differences between OR estimates [20].

#### Population attributable risk

For statistically significant associations, we estimated the population attributable risk; the proportion of pre-eclampsia cases in the population that could be attributed to the PAI-1 (−675) genotype. We calculated the population attributable risk using the formula [21]:

- 100×[prevalence×(OR – 1)/prevalence×(OR – 1)+1]

## Results

**Figure 1:**
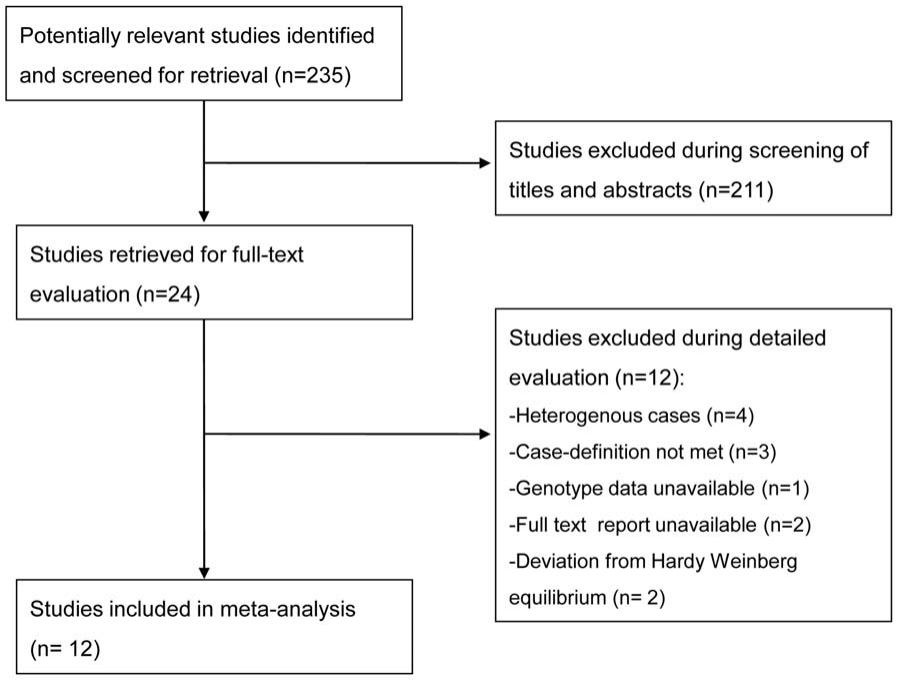
Study flow through the selection process.

Figure 1 illustrates the study selection process. Following screening, we selected 24 articles for full text assessment [22]–[45]. 12 studies met all inclusion criteria and were included in the meta-analysis [22]–[33].

### Characteristics of excluded studies

We excluded 12 studies. Four studies recruited cases with a number of different pregnancy complications and pre-eclampsia subgroup data were not available from the report or from contact with the investigators [34]–[37]. Three studies were excluded because participants were women with (non-proteinuric) gestational hypertension rather than pre-eclampsia [38]–[40]. One was excluded because genotype data were not available either from the published report or from the study investigators [41]. Two studies were excluded because of substantial and unexplained deviation from Hardy-Weinberg equilibrium [42], [43]. We were unable to obtain the full text article for two of the studies [44], [45]. From the abstracts, these appeared potentially relevant, but further clarification would have been needed in order to assess inclusion criteria.

### Characteristics of included studies

We included 12 studies in which a total of 1511 women with pre-eclampsia, eclampsia or HELLP syndrome and 3492 controls with uncomplicated pregnancies participated [22]–[33]. Most studies were small (number of cases range from 24 to 403). Eleven of the studies used a case-control design [22]–[32]. One was a cohort study [33]. Other study characteristics are described in table 1.

**Table 1:** Characteristics of included studies.

		Cases			Controls			
Study [ref]	Country & ethnicity	N =	Blood pressure (mmHg)	Proteinuria	N =	Pearson's χ^2^	Matching	Blinding of genotyping
Yamada [22]	Japan- Japanese	115	≥140/90	≥300 mg/24 h	210	1.32	Unclear	No
Morrison [23]	UK- Caucasian	403	Diastolic ≥90 on 2 occasions or single diastolic ≥110	≥300 mg/24 h	164	0.03	Maternal age and gestation	Yes
Pegoraro [24]	South Africa-Black Zulu speaking	151	≥160/110	3+ protein on dipstick	217	0.52	Maternal age and ethnicity	No
Hakli [25]	Finland- eastern Finnish	133	≥140/90	≥300 mg/24 h	115	0.0	Unclear	No
Fabbro [26]	Italy- Not stated	52	≥140/90	≥300 mg/24 h	80	0.05	Unclear	No
Tempfer [27]	Austria- Caucasian	24	≥160/110	≥5000 mg/24 h or 3+ on dipstick	24	0.30	Gestation and parity	No
DeMaat [28]	Netherlands- Not stated	157	≥140/90	2+ on dipstick (1 g/L)	157	1.85	Maternal age and delivery date	Yes
Gerhardt [29]	Germany- Not stated	97	≥160/110 or HELLP or eclampsia	≥5000 mg/24 h	275	1.03	Geographical area	No
Dalmaz [30]	Brazil- Caucasian (71%), rest not stated	75	≥140/90	≥300 mg/24 h	143	1.66	Maternal age and ethnicity, delivery date	No
Muetze [31]	Germany- Caucasian	102	HELLP	HELLP	102	0.0	Unclear	No
Kobashi [32]	Japan- Japanese	101	≥160/110	Not defined HELLP excluded	376	0.01	Geographical area	No
Said [33]	Australia- Caucasian	104	≥160/110	≥5000 mg/24 h	1629	1.05	Geographical area	No

#### Case definition

Cases were recruited in referral centres for women with a complication of pregnancy. All participants fulfilled internationally-recognised criteria for pre-eclampsia (sustained blood pressure ≥140/90 mm Hg with proteinuria ≥300 mg/24 h, after 20 weeks' gestation). In six studies, participation was restricted to women with severe pre-eclampsia (sustained blood pressure ≥160/110 mm Hg), eclampsia or HELLP syndrome [24], [27], [29], [31]–[33].

Seven studies were undertaken in Europe [23], [25]–[29], [31]. Participants were stated to be Caucasian in four studies and ethnicity was not described in three. Two studies recruited Japanese women [22], [32]. One study in South Africa enrolled only Black, Zulu language-speaking women [24]. One study recruited Caucasian women in Australia [33]. In a Brazilian study, most participants were Caucasian but the ethnicity of 29% of participants was not described [30].

#### Controls

All controls were women with a previous pregnancy uncomplicated by pre-eclampsia. Seven of the studies reported matching of controls to cases for factors included maternal age, gestational age at delivery, parity, ethnicity and geographical origin.

#### Genotyping

Only two of the study reports stated explicitly that genotyping was undertaken without operator knowledge of the phenotype.

### Genotype associations

#### Recessive model: 4G/4G versus [4G/5G or 5G/5G]

**Figure 2:**
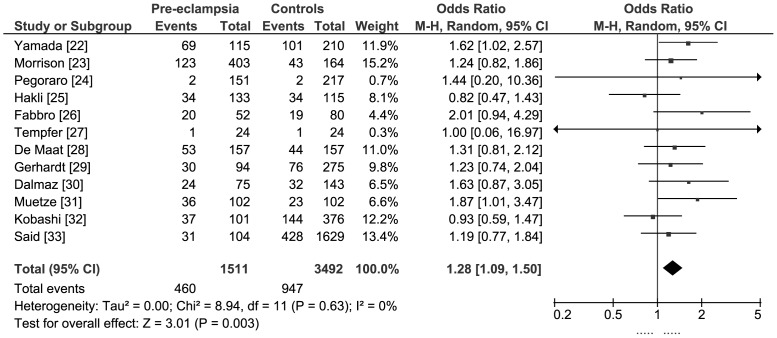
Meta-analysis of PAI-1 (−675 4G/4G) genotype and pre-eclampsia: recessive model.

Two studies found borderline statistically significant associations with pre-eclampsia [22], [31]. The remaining studies did not detect statistically significant effects. Meta-analysis of all studies found a statistically significant association between maternal carriage of the PAI-1 (-675 4G/4G) genotype and pre-eclampsia: pooled OR 1.28 (95% CI 1.09, 1.50) (Figure 2). The estimated population attributable risk for maternal carriage of the PAI-1 (−675 4G/4G) genotype was 7.1% (based on average 27% prevalence in controls).

**Figure 3:**
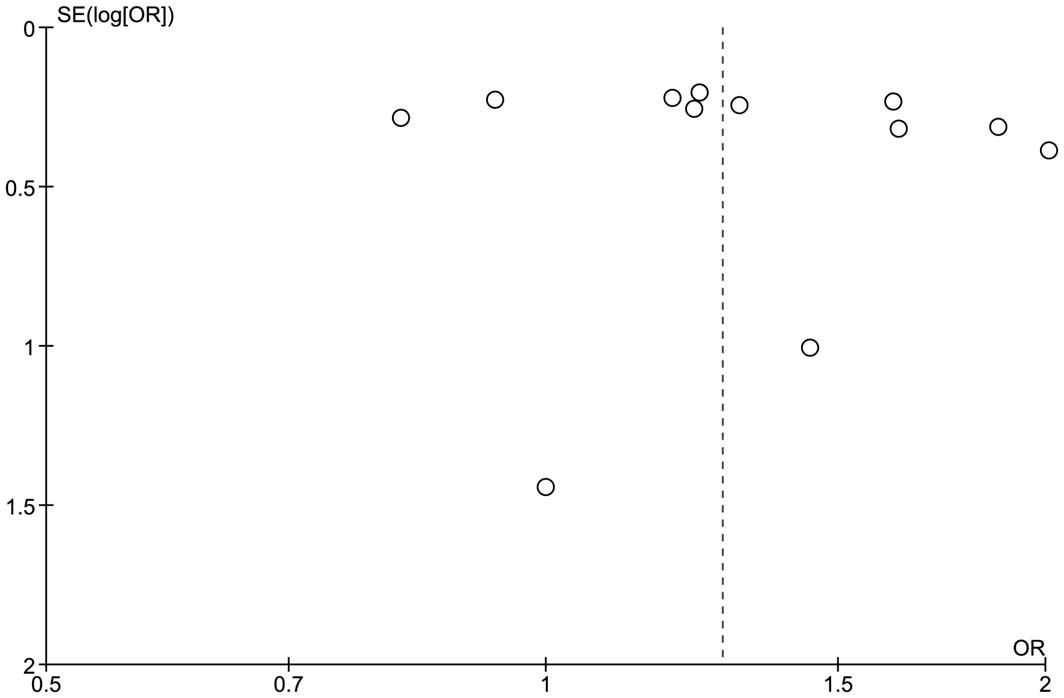
Funnel plot of PAI-1 (−675 4G/4G) genotype and pre-eclampsia.

The meta-analysis did not contain any evidence of statistical heterogeneity (I^2^ = 0%) or funnel plot asymmetry (Figure 3). Regression analysis of OR did not detect any significant linear relationships with either number of cases (co-efficient −0.001; standard error 0.001; P>0.05) or weight (co-efficient −0.03; standard error 0.03; P>0.05). In a sensitivity analysis we did not find a statistically significantly difference between the OR estimates for the six smaller versus six larger studies:

- Pooled OR for smaller studies (<103 cases): 1.34 (95% CI 1.04, 1.74)
- Pooled OR for larger studies (>103 cases): 1.24 (95% CI 1.01, 1.52)

We did not find a statistically significant difference in a post hoc subgroup analysis of six studies in which participants were women with severe pre-eclampsia, eclampsia, or HELLP syndrome (24,27,29,31–33): pooled OR 1.20 (95% CI 0.94, 1.53).

#### Dominant model [4G/5G or 4G/4G] versus 5G/5G

**Figure 4:**
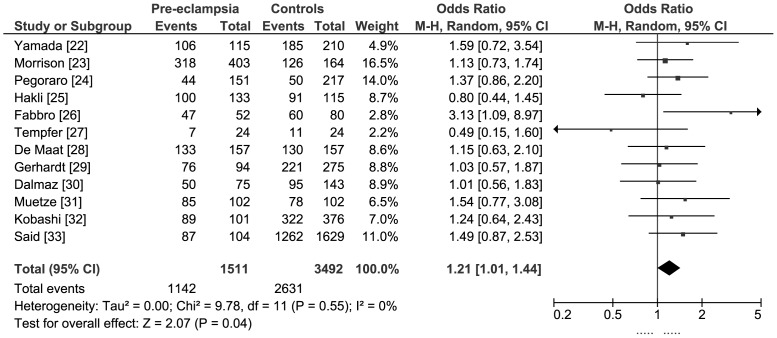
Meta-analysis of PAI-1 (−675 4G/4G or 4G/5G) genotype and pre-eclampsia: dominant model.

Only one study found a statistically significant association with pre-eclampsia [26]. Meta-analysis of data from all of the studies found a borderline statistically significant association between maternal carriage of the PAI-1 (−675 4G/5G or 4G/4G) genotypes and pre-eclampsia: pooled OR 1.21 (95%CI 1.01, 1.44) (Figure 4). The estimated population attributable risk for maternal carriage of the PAI-1 (−675 4G/4G) genotype was 13.7% (based on average 76% prevalence in controls).

**Figure 5:**
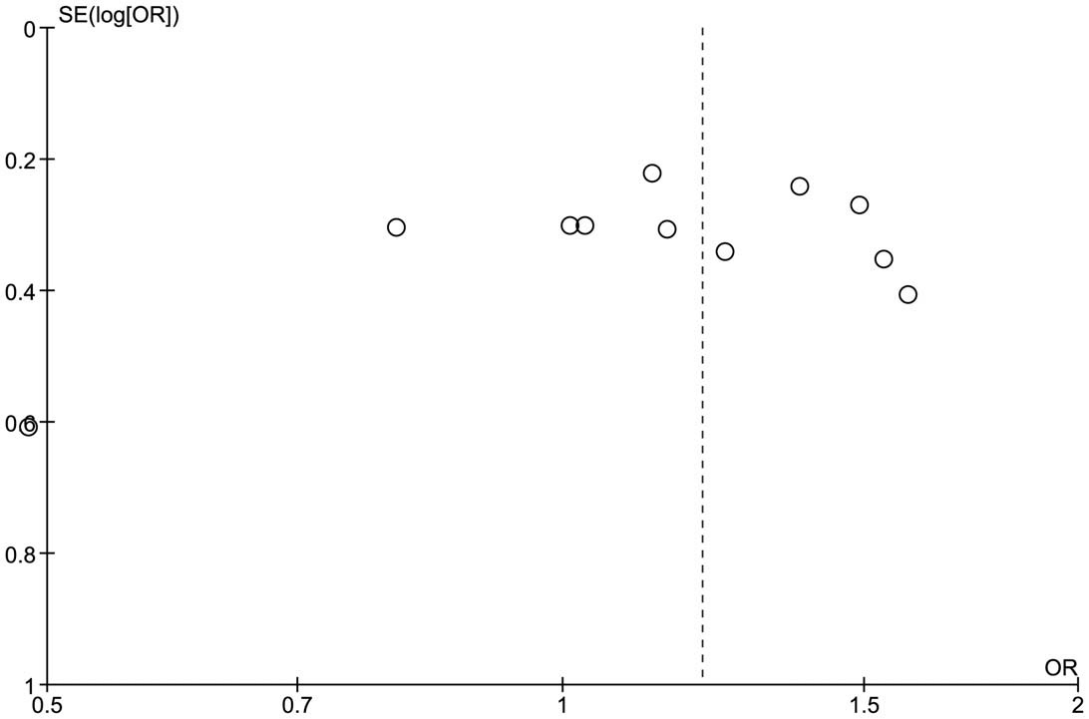
Funnel plot of PAI-1 (−675 4G/4G or 4G/5G) genotype and pre-eclampsia.

The meta-analysis did not contain any evidence of statistical heterogeneity (I^2^ = 0%) or funnel plot asymmetry (Figure 5). Regression analysis of OR did not detect any significant linear relationships with either number of cases or weight of study. There was no statistically significantly difference between the OR estimates for the six smaller versus six larger studies:

- Pooled OR for smaller studies (<103 cases): 1.20(95% CI 0.85, 1.69)
- Pooled OR for larger studies (>103 cases): 1.21 (95% CI 0.97, 1.51)

We did not find a statistically significant difference in a post hoc subgroup analysis of six studies in which participants were women with severe pre-eclampsia, eclampsia, or HELLP syndrome (24,27,29,31–33): pooled OR 1.27 (95% CI 0.99, 1.53).

## Discussion

### Summary of main results

These data suggest that the plasminogen activation and inhibition pathways may be involved causally in the pathogenesis of pre-eclampsia and related conditions. Unlike studies that report the association of plasma levels of PAI-1 with pre-eclampsia, the finding of a genetic association is not subject to “reverse causation” bias [46]. The modest strength of the association, an increase in odds of 28% in the recessive model, is consistent with reported associations of PAI-1 (−675 4G/4G) genotype with venous thrombo-embolic disease, and of other genetic variants with pre-eclampsia [47], [48]. We undertook post hoc analyses of studies that restricted participation to women with severe pre-eclampsia (blood pressure ≥160/90), eclampsia and HELLP syndrome. We did not find any evidence of a stronger genetic association as might have been expected, but this lack of evidence may be due to diminishing overall sample size.

### Implication of the findings

At the population level, the PAI-1(−675 4G/4G) genotype may account for more than 7% of all cases of pre-eclampsia with heterozygous possession of the allele accounting for a further similar proportion. However, this does not equate to an important increase in risk at an individual level. Most pregnant women who are carry the PAI-1 −675 (4G/4G) genotype will not develop pre-eclampsia and most women with pre-eclampsia do not carry the PAI-1 −675 (4G/4G) genotype. This level of association does not justify screening women for this polymorphism to target surveillance or prophylaxis for pre-eclampsia. Even if combined with other putative genetic risk factors, including those related to thrombogenesis or fibrinolysis, individual disease predictive value remains weak and imprecise compared with established clinical or demographic factors such as previous or family history of pre-eclampsia, cigarette smoking, or elevated body mass index [49]. Furthermore, the consequences of interactions between genetic thrombophilia tendencies and characteristics such as obesity and smoking that are associated both with pre-eclampsia and increased PAI-1 generation remain to be defined [1], [2].

### Limitations of the study

These findings should be regarded as preliminary and interpreted and applied cautiously. Typically, the contribution of individual genetic variants to complex conditions such as pre-eclampsia is small with OR of association generally less than 1.3 [50]. Very large studies are needed to detect plausible association sizes. While meta-analysis of data from several studies can reduce the play of chance, increase power and provide more precise estimates of association size, several important sources of bias that limit validity and applicability should be considered [51].

#### Publication and language bias

As with all systematic reviews, and despite efforts to undertake a comprehensive or exhaustive search, the possibility that the included studies do not represent the true totality of evidence remains. The principal risk relates to publication bias, the tendency for studies that find statistically significant associations to be more likely to be presented, submitted or accepted for publication in conference proceedings or journals. We did not find any evidence from funnel plot inspection and statistical modeling that this had occurred. Furthermore, only two of the studies described (borderline) statistically significant associations of the PAI-1 (−675 4G/4G) genotype with pre-eclampsia [22], [31]. However, this included the first reported study [22], consistent with a phenomenon described as “winner's curse”, the tendency for the first reported study in meta-analyses of genetic association studies to overestimate the true effect size, particularly when the study includes few participants [52]. The first report of a statistically significant disease association is likely to prompt further research in the field, but the corollary is that subsequent studies tend to under-estimate effects because of inadequate sample sizes.

Local literature bias is another potential problem [53]. For this reason, we did not use a language filter in searching the major electronic databases of publications. We were unable to obtain the full text of two Bulgarian language reports in order to translate and evaluate for inclusion [44], [45]. However, the abstracts of both of these studies described associations between maternal PAI-1 (−675 4G/4G) genotype and pre-eclampsia or related pregnancy complications with reported OR of 2.6 and 1.4 [44], [45]. Had these studies been eligible, it is likely that their inclusion in a meta-analysis would have increased size of the pooled estimate of the association.

It is also possible that we have not identified studies that are indexed only in non-English language databases. We were not able to search Chinese language databases. Many Chinese genetic epidemiology studies are published in Chinese language journals and indexed only in Chinese databases. However, evidence exists that the genetic association effect sizes reported in Chinese language studies are generally larger than those published in English language studies (“inverse language bias”) [53]. Systematic reviews should balance the need to include all studies against the risk of introducing large bias from selectively published or reported studies [51].

#### Small study bias

The related phenomenon of small-study bias (studies involving few participants being less reliable and more likely to produce false-positive results) is also well described in the context of meta-analysis of genetic epidemiology studies [52]. Some evidence exists that small study bias explains proposed links between other genetic variants, including thrombophilic mutations such as the Factor V Leiden, and pre-eclampsia [13], [54]. We did not find any evidence of a diminishing OR estimate as study size or weight increased in a linear regression model. We did not find any significant differences in the pooled OR estimates when comparing the smaller with the larger studies in a post hoc analysis. However, with only 12 studies to include in these exploratory models, small study bias remains a possibility.

#### Study design

We included both case-control and cohort studies in the meta-analyses as recommended by the Human Genetic Epidemiology Network [15]. Eleven of the studies used a traditional case-control design and one was a prospective cohort study [33]. Some concern exists that case-control studies are more subject to various sources of bias including publication bias, small study bias, reporting bias, genotyping errors, and population admixture than cohort studies [55]. Recent systematic reviews and meta-analyses of genetic epidemiology studies examining the association between carriage of the Factor V Leiden mutation and pre-eclampsia found that larger effect sizes in case-control studies than population based cohort studies [55], [56]. The single cohort study that we identified found an association size consistent with the pooled estimate from the case-control studies [33]. Given that pre-eclampsia is an uncommon condition at the population level, even large cohort studies will include few affected individuals and the comparison groups in nested case-control samples will also be small [57]. Efforts to undertake much larger studies to obviate this problem would require multicentre and multinational collaborative networks.

#### Reporting bias

Another important source of bias is within-study selective reporting. Many genetic association studies analyse the link between pre-eclampsia and several different genetic polymorphisms [2]. Selective reporting occurs when multiple analyses have been carried out but only selected subsets are reported. Authors are more likely to choose to report their findings if they reach statistical significance. One of the excluded studies appeared to examine the association of pre-eclampsia with several polymorphisms including PAI-1 (−675 4G/5G). The report stated that a statistically significant association was not found but did not provide any data [46]. This form of selective reporting bias may also exist undetected in other studies.

#### Ethnic heterogeneity

The prevalence of the PAI-1 (−675 4G/5G) polymorphism varies between ethnic groups and ethnicity affects the risk of pre-eclampsia [58]. Therefore, different ethnic distributions of cases and controls may distort any gene-disease associations. Although most of the studies in this review recruited cases and controls from ethnically-similar populations in a defined geographical region, the use of broad ethnic group categorisations and inconsistencies in how ethnicity data were defined and reported may have caused residual confounding. The possibility that population admixture has distorted the strength of the pooled estimate of association remains. Family-based studies using transmission disequilibrium testing to account for population admixture have not yet examined the association of the PAI-1 (−675 4G/5G) polymorphism with pre-eclampsia [59], [60].

#### Study quality

A major limiting factor in determining the validity and applicability of the findings of any systematic review and meta-analysis is the quality of the included studies. However, unlike the quality assessment of randomised controlled trials, empirical evidence to determine the degree to which different genetic association study characteristics introduce bias is lacking [57]. Furthermore, the methods used in many genetic association studies are reported variably and incompletely [61].

The potential methodological design weakness in genetic association studies include imprecise phenotype definition (selection bias), inappropriate control selection, and lack of blinding of laboratory genotyping staff to the clinical status of cases and controls (observer bias) [15]. We specifically excluded studies in which participants included women with gestational hypertension in order to improve the homogeneity of phenotype between studies. The absence of statistical heterogeneity in the meta-analyses is reassuring and may reflect the a priori requirement for studies to have used an internationally-accepted case definition. Furthermore, only studies in which controls were women who had a pregnancy uncomplicated by pre-eclampsia were eligible for inclusion. This inclusion criterion reduces the risk that any observed association is due to another unrelated or unknown factor.

Only two studies included in this review reported blinding of genotyping. Some of the older genotyping techniques are more subjective than the modern methods and knowing the clinical status of the case or the control may have influenced the interpretation of the genotyping result. We have managed this problem to some extent by excluding studies in which the genotype distribution within the controls deviated substantially form Hardy Weinberg equilibrium [62]. Both of the excluded studies reported very strong associations of PAI-1 (−675 4G/4G) genotype and pre-eclampsia and their inclusion would have inflated the pooled estimate in meta-analyses [42], [43].

## Conclusions

Although several potential source of bias cannot be excluded, the finding of an association with maternal carriage of the PAI-1 (−675 4G/5G) polymorphism suggests that plasminogen activation and inhibition pathways may be involved in the aetio-pathogenesis of pre-eclampsia. This genetic association does not justify screening pregnant women for carriage of PAI-1 (−675 4G/5G) polymorphism but may contribute to our understanding of the complex the molecular mechanisms underlying this condition, inform research to develop novel interventions, or help prioritise therapeutic targets that merit evaluation in randomised clinical trials.

## Supporting Information

Checklist S1 **PRISMA form.**
(DOC)
